# Mean Platelet Volume and Platelet Volume Distribution Width in Canine Parvoviral Enteritis

**DOI:** 10.3389/fvets.2021.722280

**Published:** 2021-10-06

**Authors:** Monique Engelbrecht, Brogan Atkinson, Amelia Goddard, Paolo Pazzi, Vanessa McClure

**Affiliations:** Department of Companion Animal Clinical Studies, Faculty of Veterinary Science, University of Pretoria, Pretoria, South Africa

**Keywords:** parvoviral enteritis, gastrointestinal system (GIS), platelet indices, mean platelet volume, platelet volume distribution width

## Abstract

Bacterial translocation from the damaged intestinal tract, reported in canine parvoviral (CPV) enteritis, is thought to be responsible for the systemic inflammatory response resulting from coliform septicemia, which could ultimately progress to septic shock and death. Alterations in platelet indices, specifically mean platelet volume (MPV), is a consistent finding in critically ill people and dogs with and without sepsis. Increased MPV has been reported to be an indirect indicator of platelet activation and of bone marrow response in people and dogs with sepsis. The study aim was to compare admission MPV and platelet volume distribution width (PVDW) in dogs with CPV enteritis to that of healthy aged-matched control dogs. Forty-eight dogs with CPV enteritis and 18 healthy age matched control dogs were included. CPV infection was confirmed with electron microscopy and concurrent blood-borne infections were excluded using PCR. EDTA whole blood samples were analyzed on an automated cell counter, ADVIA 2120, within 30-60 min from collection. There was no significant difference for platelet count between the groups. The MPV for CPV infected dogs (median: 14.0; *IQR*: 12.2–15.1) was significantly higher compared to controls (11.3; *IQR*: 10.3–13.1, *P* = 0.002). The PVDW for CPV infected dogs (66.9; *IQR*: 64.2–68.8) was significantly higher compared to controls (63.3; *IQR*: 60.2–65.1, *P* < *0.001*). These findings suggest that significant platelet activation is present in dogs with CPV enteritis which may play a role in the disease outcome, similar to people with sepsis. Further studies are required to investigate the prognosticating ability of MPV in dogs with CPV enteritis.

## Introduction

Canine parvovirus (CPV) is an important pathogen worldwide and a significant cause of viral enteritis in dogs with associated high morbidity and mortality rates (1). In susceptible populations, the viral infection most commonly manifests as a severe systemic and often life-threatening illness (2). Systemic inflammation may occur in many cases due to bacterial translocation from the damaged intestinal tract with a subsequent bacteremia and endotoxemia (3). This can ultimately progress to septic shock and death in severe cases (3). Endotoxins, as well as proinflammatory cytokines, are powerful mediators of the systemic inflammatory response as well as triggers of the coagulation cascade (4, 5). C-reactive protein (CRP), a major acute phase protein in dogs, is considered an accurate indicator of systemic inflammation (6). Increased serum concentrations of CRP in CPV infected dogs has been shown to be associated with disease severity and outcome (7).

Platelets are more than cytoplasmic fragments that participate in hemostasis, but have been reported to play a significant role in the host inflammatory response (8). Platelets normally circulate in blood in a resting state. Platelet activation, resulting in changes that lead to platelet adhesion and aggregation, only occur after stimulation with one of several physiological agonists, for example weak agonists such as adenosine diphosphate or stronger agonists such as thrombin or vessel wall collagen (8). Platelet indices such as mean platelet volume (MPV), mean platelet mass (MPM), mean platelet component concentration (MPC), and platelet volume distribution width (PVDW) are recognized as surrogate markers of platelet activation (8, 9). Optical based automated hematology analyzers are able to accurately measure platelet size and content and record various of these platelet indices that may serve as surrogate markers for platelet activation (8–10). Reasons for an increased number of circulating large platelets during inflammation may be twofold: (1) increased cytokine concentrations contribute to megakaryocytosis, resulting in the release of larger immature platelets regardless of the platelet concentration (11, 12); and (2) following activation, platelets undergo a shape change with small protuberances appearing on their surface, resulting in an increase in the platelet volume (10, 13).

Multiple studies in humans have investigated the utility of platelet indices, specifically those that serve as surrogate markers for platelet activation, in various inflammatory conditions; however, there is a scarcity of studies in animals. An increase in MPV has been reported in people with acute appendicitis (14), pancreatitis (15), infectious endocarditis (16), myocardial infarction (17), and malaria (18). Numerous studies in critically ill people, with sepsis, have concluded that an increased MPV at admission, as well as at multiple time points during hospitalization, indicate a poorer prognosis (19–22). In dogs with surgically treated septic peritonitis, an increase in MPV within the first 24 h of diagnosis was the only platelet parameter associated with an increased risk of mortality. The increased MPV suggested platelet activation related to the presence of a marked inflammatory response (23). In a canine model of endotoxemia, an increase in both MPV and PVDW have shown potential value in the diagnosis and monitoring of dogs with endotoxemia (24).

The objective of this study was to compare the differences in platelet indices at admission between dogs with CPV enteritis and healthy control dogs. A secondary objective was to determine whether there is a correlation between the degree of inflammation, based on the serum CRP concentration, and the platelet indices at admission. We hypothesized that the platelet indices, specifically MPV, PVDW, and MPM would be increased, whereas MPC would be decreased in dogs infected with CPV compared to that of healthy controls and that these changes would correlate with the degree of inflammation.

## Materials and Methods

### Study Design

This was a prospective, case-controlled observational study on juvenile dogs naturally infected with CPV enteritis that presented to the Onderstepoort Veterinary Academic Hospital (OVAH), over a period of 11 months between November 2018 and September 2019. Ethical approval was obtained from the University of Pretoria's research and animal ethics committees (REC065-18 and V073-18, respectively).

### Study Population

Client owned dogs diagnosed with CPV enteritis that presented to the OVAH, Faculty of Veterinary Science, University of Pretoria were included in the study. A control group of apparently healthy dogs that presented for routine vaccination or elective surgical procedures, such as orchidectomy or ovariohysterectomy, were also included. A consent form was signed by each owner prior to inclusion in the study. Inclusion criteria for the CPV infected dogs: dogs of either breed or sex, between the ages of 8 weeks and 12 months, weighing more than 3 kg and demonstrating clinical signs associated with CPV infection. Initial diagnosis of CPV enteritis was based on a dog with clinical signs consistent with CPV enteritis and a positive result from a valid, rapid patient-side immunoassay (IDEXX Laboratories©, Westbrook, USA and Bionote Inc., Gyeonggi-do, Korea). Infection was then confirmed in every case by visualization of CPV-2 through fecal electron microscopy (EM). Criteria for exclusion included treatment with any medication known to interfere with platelet number and function including prednisolone, aspirin, or non-steroidal anti-inflammatory drugs either at presentation or within 4 weeks prior to presentation. The dogs had to be free of any obvious other inflammatory processes that could falsely affect the variables. Dogs that had received any other medication or had been previously hospitalized for the treatment of CPV enteritis were also excluded. The healthy controls were age-matched to the CPV group. The control dogs were considered healthy based on history provided by the owner, physical examination as well as a normal peripheral blood smear, complete blood count (CBC), and negative fecal EM.

### Sample Collection and Laboratory Methods

At presentation, prior to any treatment, peripheral blood was collected into serum and EDTA vacutainer tubes (BD Biosciences, New Jersey, United states) from the jugular vein with a 21G needle, by careful venipuncture with minimum stasis to minimize in *vitro* platelet activation. The anti-coagulated EDTA sample was used to perform a blood smear on all dogs and was evaluated for any platelet aggregation that may falsely affect the results. The anti-coagulated EDTA sample was also used to perform the CBC, which included the platelet indices, (PLT, MPV, PDW, PCT, MPM, MPC, and PCDW) on the ADVIA 2120 (Siemens, Munich, Germany) within 30 minutes of collection. The ADVIA 2120 is an automated hematology analyzer with multispecies system software (version 6.9.0), including dogs. Serum samples were left to clot at room temperature and then centrifuged at 1,520 g for 8 min. The sample was used to determine the CRP concentration and the remaining serum was stored at−80°C. The CRP was measured using a canine specific immunoturbidimetric CRP method (Gentian, AS, Moss, Norway). A fecal sample was collected at presentation. The fecal sample was refrigerated once collected and submitted for fecal EM within 12 h of collection to be examined for the presence of CPV.

### Statistical Analysis

Statistical analysis was performed using a commercial software package (SPSS Statistics version 24^®^, IBM, New York, NY, USA). The Shapiro Wilk test was used to assess data for normality. The data had a non-normal distribution and comparison of all variables between the CPV group and the control group was analyzed using the Mann-Whitney U test. Breed and sex proportions between groups were assessed using the Chi square test. Correlation between CRP and platelet indices were determined using the Spearman's rank correlation coefficient. The level of significance was set at *P* < 0.05. Data is represented as median and interquartile range (IQR).

## Results

### Study Population Characteristics

The study comprised of 48 CPV infected dogs and 18 apparently healthy control dogs. For the CPV group 14 (29%) were female and 34 (71%) were male and of the control dogs 6 (33%) were female and 12 (66%) were male. There was no significant difference in sex ratio between the groups. The median age was 3.5 months and 4 months for the CPV infected group and control group, respectively, with no significant difference. Breeds included in the CPV group were mixed breed (15), American Pitbull terrier (8), Labrador retriever (6), Jack Russel terrier (5), Boerboel (3), boxer (2), Yorkshire terrier (2) and one each of the following breeds: basset hound, dachshund, Pekingese, Rottweiler, Saint Bernard, schnauzer, and Siberian husky. Breeds included in the control group were Boerboel (4), dachshund (2), golden retriever (2), Siberian husky (2) and one each of the following: bull terrier, Doberman pinscher, fox terrier, Jack Russel terrier, Labrador retriever, Pekingese, Scottish terrier, Staffordshire bull terrier.

### Comparison of the Platelet Indices Between CPV Infected Dogs and Healthy Controls

**Figure 1:**
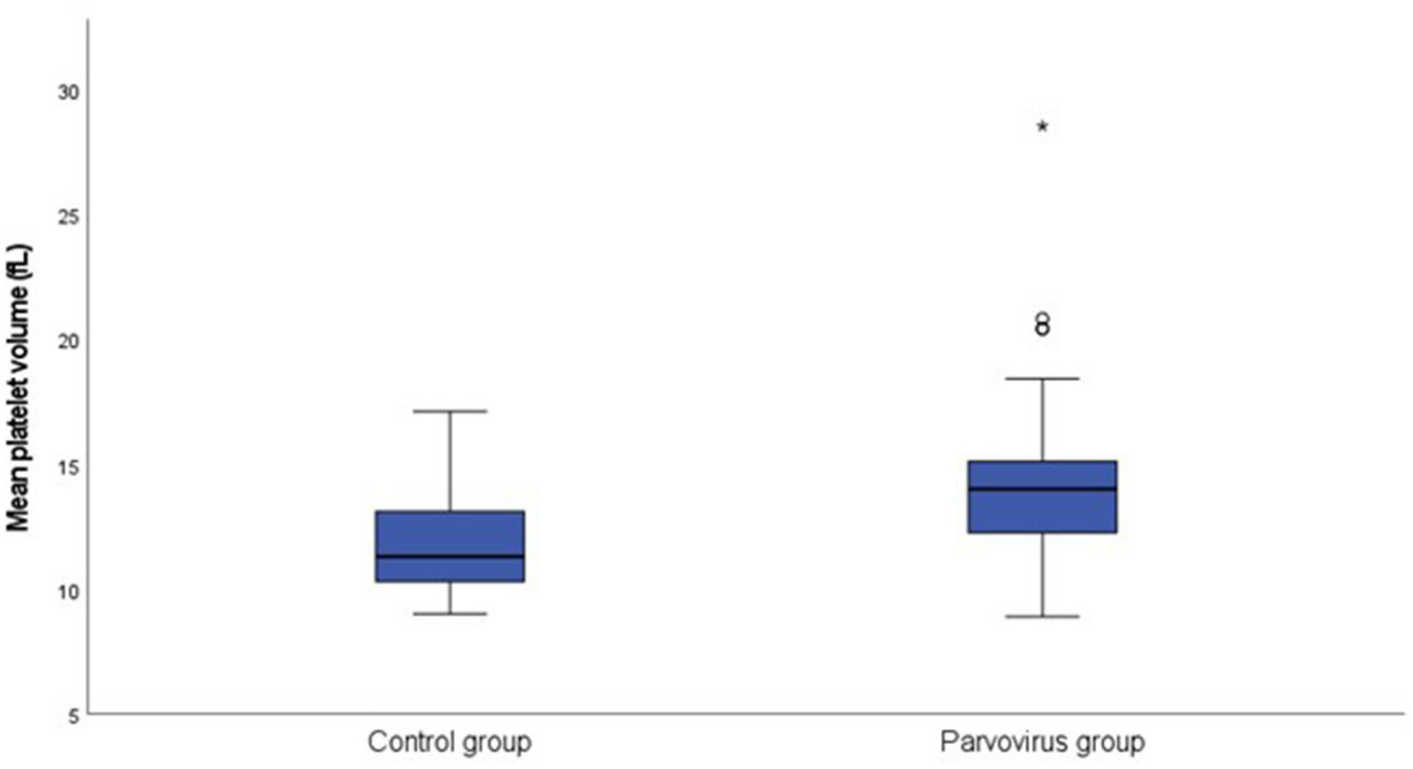
Box plot of the admission MPV of the CPV infected group (*N* = 48) compared to the healthy control group (*N* = 18). All values below the 10^th^ percentile and above the 90^th^ percentile are plotted separately as dots and the asterisk.

**Figure 2:**
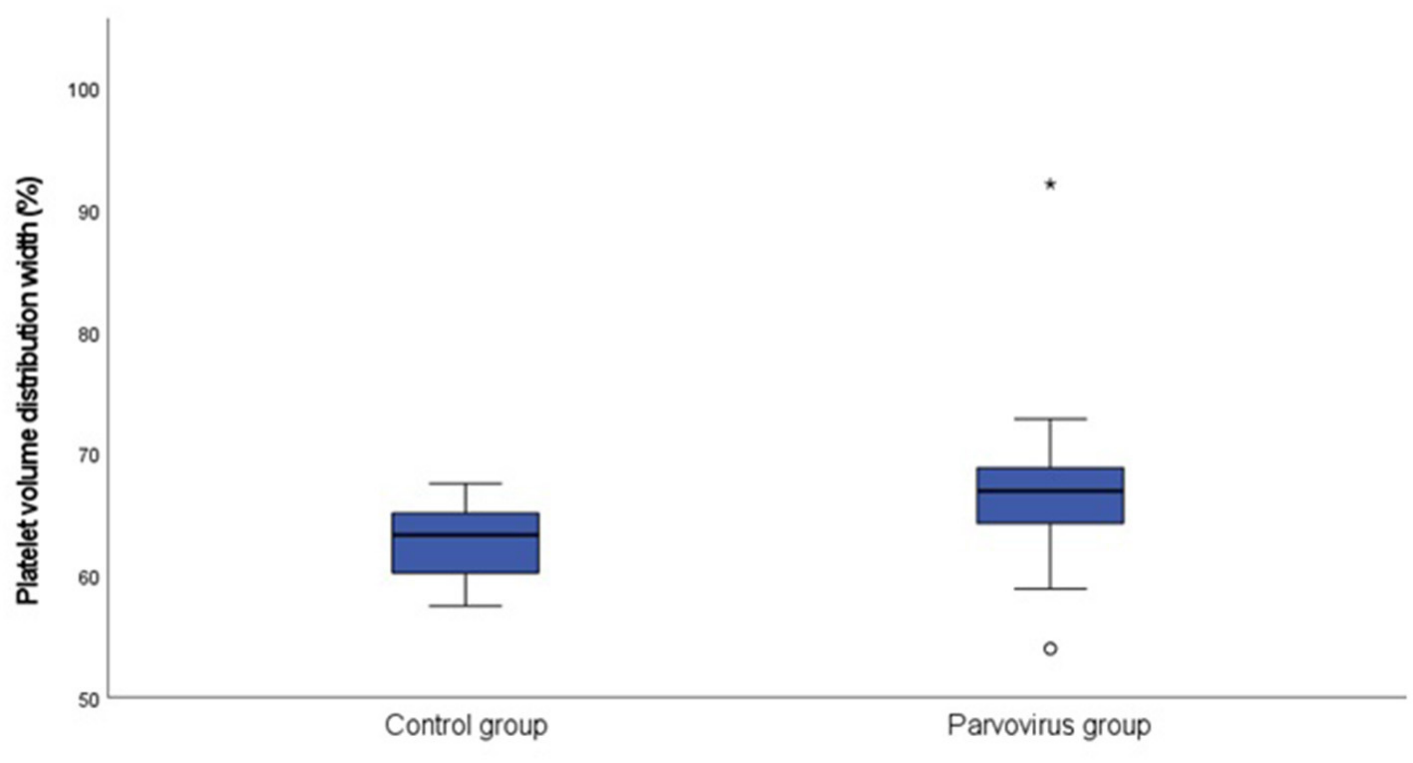
Box plot of the admission PVDW of the CPV infected group (*N* = 48) compared to the healthy control group (*N* = 18). All values below the 10^th^ percentile and above the 90^th^ percentile are plotted separately as dots and the asterisk.

Table 1 contains a summary of the platelet concentration and indices, as well as the serum CRP concentration at presentation. There was no significant difference for platelet concentration between the groups. The MPV (*P* = 0.002; Figure 1) and PVDW (*P* < 0.001; Figure 2) were significantly higher in the CPV infected group compared to the healthy control group. No significant differences were seen for the remaining indices between the CPV and control groups. The serum CRP concentration was significantly higher in the CPV group compared with the healthy control group (*P* < 0.001). There were no significant correlations between serum CRP concentration and platelet concentration or any of the platelet indices.

**Table 1:** Platelet indices and CRP concentration at presentation for dogs with CPV enteritis and healthy controls.

**Variable**	**Control**	**CPV**
	**Median (IQR)**	**Median (IQR)**
Platelet concentration (x10^9^/L)	416 (291–554)	359 (246–446)
Plateletcrit (L/L)	0.49 (0.40–0.63)	0.49 (0.34–0.64)
^*^Mean platelet volume (fL)	11.3 (10.23–13.25)	14.00 (12.23–15.10)
^*^Platelet volume distribution width (%)	63.30 (60.03–65.20)	66.90 (64.18–68.80)
Mean platelet component concentration (g/dL)	21.90 (19.58–23.63)	20.65 (19.20–22.40)
Mean platelet mass (pg)	2.26 (2.05–2.38)	2.34 (2.06–2.61)
C-reactive protein (mg/L)	3.03 (0–8.04)	150.35 (90.40–195.86)

* *Significantly higher in CPV infected dogs compared to controls*.

## Discussion

This study showed that dogs with CPV enteritis had significantly larger platelets compared to healthy dogs, despite the absence of thrombocytopenia. This finding may suggest platelet activation secondary to the marked inflammatory host response; however, there was no correlation with the degree of inflammation as measured by serum CRP concentration.

Septic shock is a common consequence of various disease conditions affecting people and animals worldwide and is commonly encountered in CPV infected dogs (25–27). Parvo viral infection results in damage to the intestinal tract which increases the risk of bacterial translocation and subsequent coliform septicemia which could eventually progress to systemic inflammation, septic shock and death (3). While thrombocytopenia is frequently reported in humans with sepsis (28–32), our study did not show a difference for the platelet concentration between the CPV infected dogs and healthy control dogs. Systemic inflammation and/or sepsis are known to affect platelets, and it has been reported that platelet activation and coagulation can occur in the initial phase of sepsis (20). Thrombopoietin, a hematopoietic cytokine, is largely responsible for the activation of megakaryocytes and the enhanced release of platelets (33). With sustained inflammation the increase in pro-inflammatory cytokines, mainly interleukin-6 (IL-6), leads to an increased production of thrombopoietin, release of large immature platelets and subsequently an increase in the circulating platelet concentration (34–37). During systemic inflammation, the increased concentrations of thrombopoietin as well as other pro-inflammatory cytokines also contribute to increased megakaryocytosis (11, 12). As a result, systemic inflammation results in an increase in the number and size of platelets regardless of the platelet count.

In our study, the MPV and PVDW was significantly higher in the dogs with CPV enteritis compared to the healthy control dogs. In contrast, a recent study reported no significant differences between dogs with parvo enteritis and healthy control dogs for both MPV and PDW (38). This however was a retrospective study and only included 26 dogs with CPV which could have resulted in a type II error. Mean platelet volume is a gauge of the average platelet size and indicates changes in either the level of platelet activation or the rate of platelet production (39). Platelet volume distribution width is an indicator of the heterogeneity of platelet size and an increase is suggestive of a large range of platelet sizes due to swelling, destruction, and immaturity (19, 40). As platelets become activated, they become spherical, small blebs appear on their surfaces, and several bioactive substances are released (10). With this transformation from quiescent disks to swollen spheres there is a subsequent increase in MPV (41). An increase in MPV has been described to be an indirect sign of alterations in platelet production and activity in humans with sepsis (20) and dogs with inflammation (42). Platelet volume indices are therefore considered early indicators of platelet activation that is regulated by inflammatory processes (43). Bacterial endotoxin is generally considered to be the principal component that results in the initiation of the systemic inflammatory response and septic shock (44). Endotoxin, an essential component present in the outer membrane of gram-negative bacteria, are responsible for many of the pathophysiologic outcomes occurring during sepsis (44, 45). Circulating endotoxin is a powerful stimulus for the initiation of coagulopathy, mediated through the initiation of inflammatory cytokine production by mononuclear phagocytes (4, 46). In a study where dogs received intravenous *Escherichia coli* endotoxin, blood was collected before inoculation and at multiple time points thereafter. Both MPV and PVDW increased by a mean of 28 and 45%, respectively, in as little as 30 min post intravenous injection and remained increased over baseline for 24 h (24). In a similar study that aimed to evaluate the effects of low dose endotoxin on platelet indices in dogs, MPV was significantly increased at 3 h compared to baseline (47). Both these studies suggest a change in platelet production and activation in septic dogs. Endotoxin has been demonstrated to be present in measurable quantities in dogs with CPV enteritis (48). Therefore, endotoxin and pro-inflammatory cytokines, both of which are fundamental to the pathophysiology of CPV enteritis are powerful mediators of inflammation and can lead to systemic activation of hemostasis (4, 49).

Large immature platelets, as demonstrated by an increase in MPV, are considered more functionally active, produce more prothrombotic factors, and are more likely to aggregate (20, 39, 50). Larger platelets have been shown to have more exposed fibrinogen receptors than smaller ones indicating a greater degree of activation and therefore an elevated risk of thrombus formation (51, 52). Increased fibrinogen concentration, secondary to the marked host inflammatory response, has been reported in dogs with CPV enteritis (53). In another study investigating the presence of hypercoagulability in dogs with CPV enteritis, there was a high prevalence of clinical thrombosis or phlebitis in these cases (54). Affected dogs had decreased antithrombin activity and thromboelastographic findings supportive of hypercoagulability, specifically an increase in the maximum amplitude (MA) (54). The MA reflects clot strength and is influenced by several factors including fibrin and fibrinogen concentration, platelet count and activity, thrombin concentration, factor XIII and hematocrit (55). Therefore, it is likely that these alteration in platelet indices, in particular MPV, together with the increased fibrinogen concentration, may contribute to the hypercoagulable state in CPV infected dogs and result in an increased risk of thrombosis as previously reported.

In our study CPV infected dogs had a significantly higher serum CRP concentration than that of the control dogs. C-Reactive protein is classified as a major positive acute phase protein in dogs due to the magnitude of its response during acute systemic inflammation (6, 56). One study reported that the mean admission CRP concentration was significantly higher in dogs with CPV enteritis that died compared to those dogs that survived (7). Surprisingly, there was no correlation between the serum CRP concentration and any of the platelet indices in our study. Serum inflammatory cytokines, as well as markers of endothelial activation, were not assessed in our study and therefore a correlation between the host inflammatory response and platelet activation/ regeneration cannot be ruled out.

There were some limitations to this study. Firstly, the small study sample size. Secondly, analyses of CPV infected dogs was based on a single baseline determination. Disease severity and duration of illness could not be standardized, and the investigators had no control over when during the course of the disease process the dogs were presented and diagnosed with CPV. Future studies, including daily determination of platelet indices may be more insightful into the host response during CPV enteritis. Related to this, the vaccination status of many dogs was unknown, which may have influenced the findings.

In conclusion, dogs with CPV enteritis in this study population had significantly higher MPV and PVDW values than control dogs at presentation. Mean platelet volume and PVDW are important variables in the diagnosis and monitoring of sepsis in humans. These parameters are accessible, inexpensive, and routinely used laboratory tests. The results of this study suggest that the investigation of MPV and PVDW as prognostic indicators in CPV infected dogs should be investigated further. Platelet indices could be evaluated in conjunction with published variables proven to be of value in prognostication.

## Data Availability Statement

The original contributions presented in the study are included in the article/Supplementary Material, further inquiries can be directed to the corresponding authors.

## Ethics Statement

The animal study was reviewed and ethical approval was obtained from the University of Pretoria's Research and Animal Ethics Committees (REC065-18 and V073-18, respectively). Written informed consent was obtained from the owners for the participation of their animals in this study.

## Author Contributions

ME: study design, data, result analysis, and primary author of the manuscript. BA: data collection and manuscript editing. AG: study design, data, result analysis, and manuscript editing, PP: data and result analysis and manuscript editing. VM: manuscript editing. All authors contributed to the article and approved the submitted version.

## Funding

This research was funded by Health and Welfare Sector Education and Training Authority postgraduate bursary.

## Conflict of Interest

The authors declare that the research was conducted in the absence of any commercial or financial relationships that could be construed as a potential conflict of interest.

## Publisher's Note

All claims expressed in this article are solely those of the authors and do not necessarily represent those of their affiliated organizations, or those of the publisher, the editors and the reviewers. Any product that may be evaluated in this article, or claim that may be made by its manufacturer, is not guaranteed or endorsed by the publisher.

## References

[1] Bird L Tappin S. Canine parvovirus: where are we in the 21st Century? Companion Animal. (2013) 18:142–6. 10.12968/coan.2013.18.4.142

[2] Smith-Carr S Macintire DK Swango LJ. Canine parvovirus. I Pathogenesis and vaccination. The Compendium on continuing education for the practicing veterinarian (USA). (1997).

[3] Goddard A Leisewitz AL. Canine parvovirus. Vet Clin North Am Small Anim Pract. (2010) 40:1041–53. 10.1016/j.cvsm.2010.07.007 20933134

[4] Weiss DJ Rashid J. The sepsis-coagulant axis: a review. J Vet Intern Med. (1998) 12:317–24. 10.1111/j.1939-1676.1998.tb02129.x 9773406

[5] Wessels BC Gaffin SL. Anti-endotoxin immunotherapy for canine parvovirus endotoxaemia. J Small Anim Pract. (1986) 27:609–15. 10.1111/j.1748-5827.1986.tb03760.x

[6] Cerón JJ Eckersall PD Martínez-Subiela S. Acute phase proteins in dogs and cats: current knowledge and future perspectives. Vet Clin Pathol. (2005) 34:85–99. 10.1111/j.1939-165X.2005.tb00019.x 15902658

[7] McClure V Van Schoor M Thompson PN Kjelgaard-Hansen M Goddard A. Evaluation of the use of serum C-reactive protein concentration to predict outcome in puppies infected with canine parvovirus. J Am Vet Med Assoc. (2013) 243:361–6. 10.2460/javma.243.3.361 23865878

[8] Macey MG Carty E Webb L Chapman ES Zelmanovic D Okrongly D. Use of mean platelet component to measure platelet activation on the ADVIA 120 haematology system. Cytometry. (1999) 38:250–5. 10516612 10.1002/(sici)1097-0320(19991015)38:5<250::aid-cyto8>3.3.co;2-b

[9] Goddard A Leisewitz AL Kristensen AT Schoeman JP. Platelet indices in dogs with babesia rossi infection. Vet Clin Pathol. (2015) 44:493–7. 10.1111/vcp.12306 26613563

[10] Kim HK Kim JE Ham CK Lee DS Park S Cho HI. Prognostic value of platelet indices as determined by ADVIA 120 in patients suspected of having disseminated intravascular coagulation. Int J Lab Hematol. (2008) 30:117–23. 10.1111/j.1751-553X.2007.00904.x 18333843

[11] Burstein SA Downs T Friese P Lynam S Anderson S Henthorn J. Thrombocytopoiesis in normal and sublethally irradiated dogs: response to human interleukin-6. Blood. (1992) 80:420–8. 10.1182/blood.V80.2.420.420 1627800

[12] Debili N Masse JM Katz A Guichard J Breton-Gorius J Vainchenker W. Effects of the recombinant hematopoietic growth factors interleukin-3, interleukin-6, stem cell factor, and leukemia inhibitory factor on the megakaryocytic differentiation of CD34+ cells. Blood. (1993) 82:84–95. 10.1182/blood.V82.1.84.bloodjournal82184 7686791

[13] Handagama P Feldman B Kono C Farver T. Mean platelet volume artifacts: the effect of anticoagulants and temperature on canine platelets. Vet Clin Pathol. (1986) 15:13–7. 10.1111/j.1939-165X.1986.tb00651.x 15334363

[14] Aydogan A Akkucuk S Arica S Motor S Karakus A Ozkan OV. The analysis of mean platelet volume and platelet distribution width levels in appendicitis. Indian J Surg. (2015) 77:495–500. 10.1007/s12262-013-0891-7 26730052 PMC4692884

[15] Mimidis K Papadopoulos V Kotsianidis J Filippou D Spanoudakis E Bourikas G. Alterations of platelet function, number and indexes during acute pancreatitis. Pancreatology. (2004) 4:22–7. 10.1159/000077024 14988655

[16] Gunebakmaz O Kaya MG Kaya EG Ardic I Yarlioglues M Dogdu O. Mean platelet volume predicts embolic complications and prognosis in infective endocarditis. Int J Infect Dis. (2010) 14:e982–5. 10.1016/j.ijid.2010.05.019 20851017

[17] von Ruecker A Hufnagel P Dickerhoff R Murday H Bidlingmaier F. Qualitative and quantitative changes in platelets after coronary-artery bypass surgery may help identify thrombotic complications and infections. Klin Wochenschr. (1989) 67:1042–7. 10.1007/BF01727006 2586010

[18] Chandra S Chandra H. Role of haematological parameters as an indicator of acute malarial infection in uttarakhand state of India. Mediterr J Hematol Infect Dis. (2013) 5:e2013009. 10.4084/mjhid.2013.009 23350022 PMC3552839

[19] Gao Y Li Y Yu X Guo S Ji X Sun T. The impact of various platelet indices as prognostic markers of septic shock. PLoS ONE. (2014) 9:e103761. 10.1371/journal.pone.0103761 25118886 PMC4131909

[20] Becchi C Al Malyan M Fabbri L Marsili M Boddi V Boncinelli S. Mean platelet volume trend in sepsis: is it a useful parameter? Minerva Anestesiol. (2006) 72:749–56. Available online at: https://www.minervamedica.it/en/journals/minerva-anestesiologica/article.php?cod=R02Y2006N09A0749 16871155

[21] Kitazawa T Yoshino Y Tatsuno K Ota Y Yotsuyanagi H. Changes in the mean platelet volume levels after bloodstream infection have prognostic value. Intern Med. (2013) 52:1487–93. 10.2169/internalmedicine.52.9555 23812196

[22] Van der Lelie J. Von dem Borne AK. Increased mean platelet volume in septicaemia. J Clin Pathol. (1983) 36:693–6. 10.1136/jcp.36.6.693 6343437 PMC498352

[23] Llewellyn EA Todd JM Sharkey LC Rendahl A. A pilot study evaluating the prognostic utility of platelet indices in dogs with septic peritonitis. J Vet Emerg Crit Care. (2017) 27:569–78. 10.1111/vec.12628 28749085

[24] Yilmaz Z Eralp O Ilcol YO. Evaluation of platelet count and its association with plateletcrit, mean platelet volume, and platelet size distribution width in a canine model of endotoxemia. Vet Clin Pathol. (2008) 37:159–63. 10.1111/j.1939-165X.2008.00023.x 18533914

[25] Dellinger R Carlet J Masur H Gerlach H Calandra T Cohen J. Surviving sepsis campaign guidelines for management of severe sepsis and septic shock. Intensive Care Med. (2004) 30:536–55. 10.1007/s00134-004-2210-z 14997291

[26] de Laforcade AM Freeman LM Shaw SP Brooks MB Rozanski EA Rush JE. Hemostatic changes in dogs with naturally occurring sepsis. J Vet Intern Med. (2003) 17:674–9. 10.1111/j.1939-1676.2003.tb02499.x 14529134

[27] Otto CM. Clinical trials in spontaneous disease in dogs: a new paradigm for investigations of sepsis. J Vet Emerg Crit Care. (2007) 17:359–67. 10.1111/j.1476-4431.2007.00249.x

[28] Baughman RP Lower EE Flessa HC Tollerud DJ. Thrombocytopenia in the intensive care unit. Chest. (1993) 104:1243–7. 10.1378/chest.104.4.1243 8404200

[29] Guclu E Durmaz Y Karabay O. Effect of severe sepsis on platelet count and their indices. Afr Health Sci. (2013) 13:333–8. 10.4314/ahs.v13i2.19 24235932 PMC3824485

[30] Sharma B Sharma M Majumder M Steier W Sangal A Kalawar M. Thrombocytopenia in septic shock patients–a prospective observational study of incidence, risk factors and correlation with clinical outcome. Anaesth Intensive Care. (2007) 35:874–80. 10.1177/0310057X0703500604 18084977

[31] Stephan F Hollande J Richard O Cheffi A Maier-Redelsperger M Flahault A. Thrombocytopenia in a surgical ICU. Chest. (1999) 115:1363–70. 10.1378/chest.115.5.1363 10334154

[32] Venkata C Kashyap R Farmer JC Afessa B. Thrombocytopenia in adult patients with sepsis: incidence, risk factors, and its association with clinical outcome. J Intensive Care. (2013) 1:9. 10.1186/2052-0492-1-9 25810916 PMC4373028

[33] Korniluk A Koper-Lenkiewicz OM Kaminska J Kemona H Dymicka-Piekarska V. Mean platelet volume (MPV): new perspectives for an old marker in the course and prognosis of inflammatory conditions. Mediators Inflamm. (2019) 2019:9213074. 10.1155/2019/9213074 31148950 PMC6501263

[34] Kaushansky K. The molecular mechanisms that control thrombopoiesis. J Clin Invest. (2005) 115:3339–47. 10.1172/JCI26674 16322778 PMC1297257

[35] Kuter DJ. Thrombopoietin: biology and clinical applications. Oncologist. (1996) 1:98–106. 10.1634/theoncologist.1-1-98 10387974

[36] Wolber EM Jelkmann W. Interleukin-6 increases thrombopoietin production in human hepatoma cells HepG2 and Hep3B. J Interferon Cytokine Res. (2000) 20:499–506. 10.1089/10799900050023915 10841078

[37] Kaser A Brandacher G Steurer W Kaser S Offner FA Zoller H. Interleukin-6 stimulates thrombopoiesis through thrombopoietin: role in inflammatory thrombocytosis. Blood. (2001) 98:2720–5. 10.1182/blood.V98.9.2720 11675343

[38] Koenhemsi L. Determination of platelet count and platelet indices in canine parvoviral enteritit. Med Sciand Discov. (2019) 6:24–6. 10.17546/msd.522081

[39] Bancroft AJ Abel EW McLaren M Belch JJ. Mean platelet volume is a useful parameter: a reproducible routine method using a modified coulter thrombocytometer. Platelets. (2000) 11:379–87. 10.1080/09537100020008311 11132104

[40] Bommer NX Shaw DJ Milne EM Ridyard AE. Platelet distribution width and mean platelet volume in the interpretation of thrombocytopenia in dogs. J Small Anim Pract. (2008) 49:518–24. 10.1111/j.1748-5827.2008.00636.x 18793252

[41] Laufer N Grover NB Ben-Sasson S Freund H. Effects of adenosine diphosphate, colchicine and temperature on size of human platelets. Thromb Haemost. (1979) 41:491–7. 10.1055/s-0038-1646801 462416

[42] Moritz A Walcheck BK Weiss DJ. Evaluation of flow cytometric and automated methods for detection of activated platelets in dogs with inflammatory disease. Am J Vet Res. (2005) 66:325–9. 10.2460/ajvr.2005.66.325 15757135

[43] Vagdatli E Gounari E Lazaridou E Katsibourlia E Tsikopoulou F Labrianou I. Platelet distribution width: a simple, practical and specific marker of activation of coagulation. Hippokratia. (2010) 14:28–32. 20411056 PMC2843567

[44] Horn DL Morrison DC Opal SM Silverstein R Visvanathan K Zabriskie JB. What are the microbial components implicated in the pathogenesis of sepsis? report on a symposium. Clin Infect Dis. (2000) 31:851–8. 10.1086/318127 11049761

[45] Minasyan H. Sepsis: mechanisms of bacterial injury to the patient. Scand J Trauma Resusc Emerg Med. (2019) 27:19. 10.1186/s13049-019-0596-4 30764843 PMC6376788

[46] Rocha E Paramo JA Montes R Panizo C. Acute generalized, widespread bleeding. diagnosis and management. Haematologica. (1998) 83:1024–37. Available onlinet at: https://dadun.unav.edu/bitstream/10171/22268/1/Haematologica1998_831024.pdf 9864925

[47] Flatland B Fry MM LeBlanc CJ Rohrbach BW. Leukocyte and platelet changes following low-dose lipopolysaccharide administration in five dogs. Res Vet Sci. (2011) 90:89–94. 10.1016/j.rvsc.2010.05.016 20965082

[48] Isogai E Isogai H Onuma M Mizukoshi N Hayashi M Namioka S. Escherichia coli associated endotoxemia in dogs with parvovirus infection. Nihon juigaku zasshi. (1989) 51:597–606. 10.1292/jvms1939.51.597 2548025

[49] Otto CM Drobatz KJ Soter C. Endotoxemia and tumor necrosis factor activity in dogs with naturally occurring parvoviral enteritis. J Vet Intern Med. (1997) 11:65–70. 10.1111/j.1939-1676.1997.tb00075.x 9127292

[50] O'malley T Langhorne P Elton R Stewart C. Platelet size in stroke patients. Stroke. (1995) 26:995–9. 10.1161/01.STR.26.6.995 7762052

[51] Frojmovic M Wong T. Dynamic measurements of the platelet membrane glycoprotein IIb-IIIa receptor for fibrinogen by flow cytometry. II platelet size-dependent subpopulations. Biophys J. (1991) 59:828–37. 10.1016/S0006-3495(91)82295-0 1905967 PMC1281248

[52] Jackson CW Jennings LK. Heterogeneity of fibrinogen receptor expression on platelets activated in normal plasma with ADP: analysis by flow cytometry. Br J Haematol. (1989) 72:407–14. 10.1111/j.1365-2141.1989.tb07724.x 2765407

[53] Whitehead Z Goddard A Botha WJ Pazzi P. Haemostatic changes associated with fluid resuscitation in canine parvoviral enteritis. J S Afr Vet Assoc. (2020) 91:e1–9. 10.4102/jsava.v91i0.2005 32787422 PMC7433229

[54] Otto CM Rieser TM Brooks MB Russell MW. Evidence of hypercoagulability in dogs with parvoviral enteritis. J Am Vet Med Assoc. (2000) 217:1500–4. 10.2460/javma.2000.217.1500 11128540

[55] Donahue SM Otto CM. Thromboelastography: a tool for measuring hypercoagulability, hypocoagulability, and fibrinolysis. J Vet Emerg Crit Care. (2005) 15:9–16. 10.1111/j.1476-4431.2005.04025.x

[56] Baumann H Gauldie J. The acute phase response. Immunol Today. (1994) 15:74–80. 10.1016/0167-5699(94)90137-6 7512342

