# Supplementary material for: Mean Platelet Volume and Platelet Volume Distribution Width in Canine Parvoviral Enteritis
Source: Front Vet Sci. 2021 Oct 6;8:722280. doi: 10.3389/fvets.2021.722280 (PMC8526561; doi:10.3389/fvets.2021.722280)
Supplement: Supplementary file 1 [file Data_Sheet_1.DOCX]

Supplementary Material

# Supplementary Data

Data collected from the naturally infected CPV dogs.

| **Patient** | **Patient no.** | **Age (m)** | **PLT** | **MPV** | **PDW** | **PCT** | **MPC** | **MPM** | **Retic %** | **ARC** | **CHr** | **MCVr** | **RCC** | **Ht** | **MCHC** | **CRP** | **MPXI** | **Alb** |
| --- | --- | --- | --- | --- | --- | --- | --- | --- | --- | --- | --- | --- | --- | --- | --- | --- | --- | --- |
| Parvo 1 | 7235218 | 4 | 319 | 16,2 | 66,9 | 0,52 | 18,9 | 2,36 | 5,57 | 273,6 | 25,4 | 92,5 | 4,91 | 0,35 | 322 | 190,66 | 20,3 | 23,41 |
| Parvo 2 | 7236218 | 10 | 290 | 15,5 | 68,8 | 0,45 | 20,5 | 2,53 | 0,57 | 39,8 | 23,8 | 78,9 | 6,97 | 0,46 | 346 | 195,04 | 28,6 | 29,13 |
| Parvo 3 | 7255818 | 10 | 258 | 11,9 | 61 | 0,31 | 22,7 | 2,47 | 0,37 | 34,5 | 24,2 | 77,7 | 9,45 | 0,64 | 345 | 211,23 | 25,6 | 27,93 |
| Parvo 4 | 7276218 | 9 | 222 | 9,1 | 61,3 | 0,2 | 23 | 1,92 | 0,28 | 23,7 | 22,3 | 70,8 | 8,53 | 0,55 | 360 | 204,17 | 15,9 | 26,09 |
| Parvo 5 | 7276418 | 3 | 449 | 14,2 | 64,4 | 0,64 | 18,8 | 0,82 | 2,19 | 135,9 | 26,4 | 90,4 | 6,21 | 0,45 | 342 | 83,22 | 6,5 | 36,43 |
| Parvo 6 | 7280718 | 3 | 382 | 14,6 | 66,3 | 0,56 | 19,4 | 2,29 | 0,74 | 45 | 25,1 | 81,8 | 6,11 | 0,44 | 330 | 220,35 | 12,3 | 29,73 |
| Parvo 7 | 7285618 | 12 | 163 | 10 | 70,5 | 0,16 | 22,4 | 2,04 | 0,78 | 69,6 | 22,5 | 76 | 8,93 | 0,62 | 328 | 181,38 | 17,5 | 38 |
| Parvo 8 | 7292918 | 12 | 628 | 12,9 | 72,8 | 0,81 | 19,4 | 1,98 | 2,85 | 187,3 | 25,5 | 86,4 | 6,57 | 0,5 | 391 | 366 | 14,8 | 32,34 |
| Parvo 9 | 7356918 | 4 | 361 | 11,7 | 69,4 | 0,42 | 19,5 | 1,87 | 0,81 | 55,1 | 24,1 | 79,5 | 6,85 | 0,46 | 329 | 126,39 | 11 | 32,13 |
| Parvo 10 | 7372218 | 3 | 438 | 10,5 | 63,1 | 0,46 | 22,2 | 2 | 1,34 | 71,3 | 26,7 | 85,1 | 5,3 | 0,38 | 336 | 191,27 | 17,7 | 30,8 |
| Parvo 11 | 7376018 | 4 | 323 | 18,4 | 61,1 | 0,6 | 18,3 | 2,67 | 1,48 | 81,7 | 22,6 | 73,8 | 5,52 | 0,37 | 326 | 180,53 | 16,1 | 25,05 |
| Parvo 12 | 7376918 | 2 | 584 | 14,9 | 69,7 | 0,87 | 19,2 | 2,31 | 2,85 | 161,9 | 20,3 | 69,3 | 5,69 | 0,4 | 316 | 266,3 | 14,4 | 30,49 |
| Parvo 13 | 7464419 | 2 | 386 | 14,7 | 67,7 | 0,57 | 22,6 | 2,74 | 2,04 | 121,9 | 24 | 82,4 | 5,98 | 0,37 | 309 | 98,9 | 13 | 27,54 |
| Parvo 14 | 7476719 | 3 | 270 | 13,7 | 66 | 0,37 | 22,2 | 2,6 | 0 | 0 | 0 | 0 | 3,83 | 0,25 | 323 | 91,48 | 12,5 | 25,7 |
| Parvo 15 | 7488419 | 12 | 398 | 9,2 | 64,1 | 0,37 | 23,6 | 1,99 | 0,72 | 63,9 | 24,3 | 76 | 8,92 | 0,6 | 348 | 289,52 | 17,6 | 31,47 |
| Parvo 16 | 7491019 | 2 | 1982 | 14,7 | 69,8 | 2,92 | 20,8 | 2,37 | 1,48 | 74,4 | 16,8 | 62,9 | 5,02 | 0,29 | 270 | 85,27 | 12,6 | 20,61 |
| Parvo 17 | 7495319 | 4 | 368 | 13,9 | 67,7 | 0,51 | 20,8 | 2,37 | 1,15 | 68,3 | 24,9 | 79,9 | 5,93 | 0,39 | 334 | 56,51 | 10,8 | 35,9 |
| Parvo 18 | 7528419 | 3 | 487 | 28,5 | 70,6 | 1,39 | 22,6 | 3,41 | 0,78 | 46,5 | 23,3 | 80,8 | 5,99 | 0,38 | 349 | 195 | 23,7 | 24,42 |
| Parvo 19 | 7550619 | 6 | 94 | 14,4 | 69,7 | 0,14 | 23,5 | 2,86 | 2,38 | 165,3 | 25,7 | 86,2 | 6,94 | 0,49 | 339 | 212,85 | 21,1 | 33,94 |
| Parvo 20 | 7559119 | 4 | 269 | 10,5 | 65,6 | 0,28 | 21,6 | 2,03 | 1,14 | 63 | 23,8 | 80,7 | 5,53 | 0,38 | 336 | 81,56 | 24,6 | 29,85 |
| Parvo 21 | 7568119 | 6 | 246 | 15,1 | 66,8 | 0,37 | 18,5 | 2,18 | 0,05 | 3,9 | 22 | 68,1 | 7,42 | 0,44 | 338 | 209,39 | 25,8 | 27,86 |
| Parvo 22 | 7587519 | 3 | 380 | 13,7 | 65,3 | 0,52 | 19,7 | 2,21 | 0,87 | 48,9 | 24,2 | 81 | 5,62 | 0,38 | 339 | 423,08 | 27,8 | 34,6 |
| Parvo 23 | 7607919 | 2 | 468 | 14,5 | 67,9 | 0,68 | 20,2 | 2,4 | 5,81 | 261,3 | 23,8 | 93,2 | 4,5 | 0,31 | 307 | 9 | 20,5 | 26,07 |
| Parvo 24 | 7607819 | 2 | 181 | 20,4 | 62,9 | 0,37 | 18,3 | 2,93 | 3,53 | 113 | 23,2 | 89,4 | 3,2 | 0,23 | 356 | 94,31 | 16,5 | 26,98 |
| Parvo 25 | 7637519 | 6 | 357 | 15,5 | 92 | 0,56 | 22,2 | 2,47 | 0,58 | 48,8 | 22,1 | 73,6 | 8,46 | 0,58 | 371 | 150,35 | 22,9 | 17,94 |
| Parvo 26 | 7662919 | 4 | 248 | 15,1 | 68,8 | 0,38 | 19,3 | 2,31 | 0,98 | 51,4 | 21,9 | 74,1 | 5,25 | 0,34 | 335 | 86,19 | 19 | 26,98 |
| Parvo 27 | 7663119 | 4 | 425 | 8,9 | 54 | 0,38 | 24 | 1,99 | 1,04 | 73,5 | 23,8 | 82,2 | 7,1 | 0,45 | 341 | 89,69 | 18,2 | 31,2 |
| Parvo 28 | 7673319 | 6 | 179 | 14 | 67,1 | 0,25 | 19,7 | 2,2 | 1,12 | 79,4 | 26 | 84,7 | 7,06 | 0,48 | 337 | 176,01 | 26,6 | 33,08 |
| Parvo 29 | 7713719 | 4 | 479 | 12,3 | 67,9 | 0,59 | 20 | 2,01 | 0,67 | 47 | 22,1 | 76,5 | 6,96 | 0,44 | 350 | 70,04 | 20,9 | 30,07 |
| Parvo 30 | 7790419 | 4 | 237 | 14 | 63,9 | 0,33 | 21,8 | 2,63 | 2,12 | 115,9 | 22,7 | 79,1 | 5,47 | 0,37 | 320 | 80,04 | 24,7 | 26,21 |
| Parvo 31 | 7790519 | 6 | 620 | 13 | 68,2 | 0,8 | 19,7 | 2,11 | 1,05 | 65,5 | 22,4 | 80,3 | 6,25 | 0,39 | 330 | 34,25 | 21,3 | 24,51 |
| Parvo 32 | 7851719 | 3 | 284 | 12 | 64,5 | 0,34 | 21,6 | 2,3 | 2,86 | 150 | 25,2 | 84,3 | 5,24 | 0,36 | 325 | 185,59 | 21,9 | 29,7 |
| Parvo 33 | 8101019 | 7 | 181 | 13,4 | 71,1 | 0,24 | 23,6 | 2,72 | 0,94 | 62,6 | 23,1 | 78,9 | 6,64 | 0,46 | 336 | 118,64 | 24,7 | 32 |
| Parvo 34 | 8114919 | 3 | 246 | 13,8 | 72,4 | 0,34 | 24,5 | 2,8 | 0,41 | 35,6 | 20,5 | 70,2 | 8,72 | 0,57 | 327 | 127,15 | 17,2 | 31,96 |
| Parvo 35 | 8165319 | 5 | 243 | 11,6 | 66,1 | 0,28 | 23,4 | 2,43 | 0,27 | 20,1 | 22,6 | 75 | 7,58 | 0,49 | 344 | 237,51 | 30,3 | 33,7 |
| Parvo 36 | 8169219 | 1 | 410 | 14,4 | 58,9 | 0,59 | 16,4 | 7,4 | 0,45 | 21,5 | 21,2 | 73,2 | 4,74 | 0,31 | 321 | 174,4 | 23,1 | 17,92 |
| Parvo 37 | 8170419 | 2 | 647 | 12,2 | 66,5 | 0,79 | 21,1 | 2,21 | 1,41 | 84,8 | 23,5 | 76,5 | 6,01 | 0,39 | 340 | 125,71 | 19,5 | 28,01 |
| Parvo 39 | 8281019 | 12 | 410 | 15,4 | 67,8 | 0,63 | 19,4 | 2,39 | 0,99 | 74 | 23,9 | 79,9 | 7,48 | 0,52 | 336 | 172,21 | 18,5 | 31,37 |
| Parvo P1 | 7228518 | 6 | 596 | 11,5 | 66,9 | 0,68 | 22,4 | 2,2 |  |  |  |  | 7,09 | 0,49 | 334 |  | 13,7 |  |
| Parvo P2 | 7229518 | 6 | 96 | 20,8 | 67,1 | 0,2 | 21,1 | 3,29 |  |  |  |  | 5,09 | 0,34 | 96 | 124,58 | 10,9 | 29,61 |
| Parvo P3 | 7236618 | 4,5 | 372 | 15,1 | 65,3 | 0,56 | 19,2 | 2,28 | 1,39 | 77,5 | 21,8 | 73,2 | 5,58 | 0,37 | 327 | 142,08 | 16,9 | 30,99 |
| Parvo P4 | 7237018 | 4 | 254 | 14,6 | 65,7 | 0,37 | 16,7 | 1,9 | 0,63 | 39,2 | 21,3 | 73,7 | 6,25 | 0,37 | 333 | 155,47 | 11,8 | 31,18 |
| Parvo P5 | 7239518 | 3 | 303 | 17,2 | 62,3 | 0,52 | 18,9 | 2,61 | 2,62 | 129,7 | 25,8 | 89,6 | 4,94 | 0,36 | 313 | 58,72 | 12,2 | 32,23 |
| Parvo P6 | 7249818 | 3 | 368 | 20,4 | 61,5 | 0,75 | 18,2 | 2,84 | 1,74 | 104,6 | 20 | 69,4 | 6 | 0,34 | 310 | 121,92 | 9,3 | 29,21 |
| Parvo P7 | 7265018 | 6 | 213 | 12,5 | 69,3 | 0,27 | 22,5 | 2,42 |  |  |  |  | 6,72 | 0,45 | 343 |  | 12,1 |  |
| Parvo P8 | 7265518 | 2 | 690 | 16,6 | 62,6 | 1,14 | 18,5 | 2,43 |  |  |  |  | 4,47 | 0,32 | 307 | 91,11 | 16,1 | 24,57 |
| Parvo P12 | 7388618 | 4 | 265 | 13,4 | 67,9 | 0,35 | 21,2 | 2,32 |  |  |  |  | 6,19 | 0,41 | 337 | 196,68 | 21,7 | 32,07 |
| Parvo P13 | 7388718 | 3 | 588 | 12,6 | 67,1 | 0,74 | 19,1 | 1,92 |  |  |  |  | 5,65 | 0,37 | 372 |  | 15 |  |

Data collected from the healthy control dogs.

| **Patient** | **Patient no.** | **Age (m)** | **PLT** | **MPV** | **PDW** | **PCT** | **MPC** | **MPM** | **Retic %** | **ARC** | **CHr** | **MCVr** | **RCC** | **Ht** | **MCHC** | **CRP** | **MPXI** | **Alb** |
| --- | --- | --- | --- | --- | --- | --- | --- | --- | --- | --- | --- | --- | --- | --- | --- | --- | --- | --- |
| Control 4 | 7446719 | 4 | 352 | 12,2 | 64,7 | 0,43 | 21,8 | 2,25 | 2,39 | 157,8 | 26,3 | 92,5 | 6,59 | 0,44 | 328 | 14,84 | 20,1 | 37,58 |
| Control 5 | 7677319 | 6 | 417 | 14,6 | 65,1 | 0,61 | 20 | 2,38 | 1,38 | 95,3 | 26 | 88,8 | 6,91 | 0,48 | 332 | 4,42 | 17,8 | 35,39 |
| Control 6 | 7623019 | 3 | 362 | 10,3 | 65,5 | 0,37 | 24 | 2,26 | 2,21 | 97,1 | 26,1 | 94,6 | 4,39 | 0,33 | 318 | 1,63 | 16,3 | 29 |
| Control 7 | 6887218 | 12 | 206 | 12,1 | 64,5 | 0,25 | 23,5 | 2,65 | 0,48 | 37,4 | 26 | 86,3 | 7,78 | 0,54 | 334 | 11,01 | 16,2 | 34,89 |
| Control 8 | 7512419 | 4 | 291 | 17,1 | 65,6 | 0,5 | 19,5 | 2,65 | 1,57 | 99,6 | 23,6 | 81 | 6,33 | 0,39 | 346 | 0 | 15,6 | 34,25 |
| Control 9 | 7741619 | 8 | 253 | 9,1 | 60,2 | 0,23 | 24,3 | 2,07 | 0,67 | 50 | 25,4 | 86,5 | 7,48 | 0,51 | 335 | 0 | 22,4 | 37,23 |
| Control 10 | 7741319 | 6 | 415 | 9,9 | 59,5 | 0,41 | 22 | 1,9 | 1,51 | 80,1 | 26,3 | 89,4 | 5,31 | 0,37 | 346 | 0 | 25,8 | 34,84 |
| Control 11 | 7741219 | 6 | 438 | 11 | 60,6 | 0,48 | 21,7 | 2,07 | 2,39 | 134,4 | 26,1 | 91,4 | 5,62 | 0,4 | 346 | 0 | 21,6 | 32,24 |
| Control 12 | 7756919 | 12 | 292 | 14,9 | 58 | 0,44 | 19,1 | 2,33 | 0,87 | 57 | 25,5 | 88,9 | 6,54 | 0,45 | 337 | 11 | 29 | 41,14 |
| Control 13 | 7507419 | 5 | 270 | 11,2 | 62,5 | 0,3 | 23,3 | 2,39 | 1,7 | 105,1 | 24,6 | 83,1 | 6,17 | 0,4 | 347 | 0 | 35,6 | 30,84 |
| Control 14 | 7775119 | 3 | 559 | 10,7 | 63,2 | 0,6 | 19,6 | 1,77 | 4,29 | 229,4 | 24,5 | 88,7 | 5,35 | 0,37 | 319 | 50,58 | 29,7 | 31,78 |
| Control 14b | 7746119 | 3 | 434 | 10 | 57,5 | 0,43 | 22,1 | 2,08 | 2,25 | 127,8 | 26,4 | 94,4 | 5,69 | 0,39 | 342 | 0 | 19,7 | 37,14 |
| Control 15 | 7804819 | 2 | 395 | 12,5 | 63,4 | 0,49 | 22,4 | 2,45 | 0,98 | 42,7 | 23,5 | 87 | 4,37 | 0,3 | 335 | 0 | 8,2 | 28,82 |
| Control 16 | 7730019 | 3 | 722 | 11,4 | 66 | 0,82 | 19,3 | 1,86 | 2,43 | 138,8 | 25,7 | 91,2 | 5,72 | 0,41 | 336 | 7,05 | 17,7 | 37,02 |
| Control 18 | 7858019 | 2 | 558 | 13,1 | 64,2 | 0,73 | 20,8 | 2,29 | 2,3 | 137 | 25 | 85,7 | 5,96 | 0,41 | 328 | 0 | 22,5 | 31,37 |
| Control 19 | 7858019 | 2 | 489 | 13,7 | 61,7 | 0,67 | 18,3 | 2,06 | 2,55 | 120,5 | 24,2 | 84,9 | 4,73 | 0,32 | 385 | 4,42 | 22 | 28,4 |
| Control 20 | 7892319 | 2 | 553 | 10,3 | 67,5 | 0,57 | 24,6 | 2,32 | 3,59 | 178 | 25 | 89,8 | 4,96 | 0,35 | 313 | 6,25 | 23,1 | 29,86 |
| Control 21 | 7973919 | 2 | 744 | 9 | 58 | 0,67 | 24,2 | 2,03 | 2,22 | 103,1 | 24,4 | 91 | 4,64 | 0,32 | 303 | 6,26 | 15,7 | 28,12 |
